# Transactions Medical Society of Virginia

**Published:** 1874-05

**Authors:** 


TRANSACTIONS of the Fourth Annual Session of the Medical Society of Virgina, held in Norfolk, November 11th to 14th, 1873, with an alphabetical register of fellows, Richmond. Fergusson & Rady, 1874. Octavo, paper, pp. 124-

In the appendix are to be found, Annual Address of the President, on the duties of this Society and the State, regarding Irregular Practitioners, and Adulterated Medicines, by Harvey Black, M.D., Blacksburg, Va.; Annual Address on the Reciprocal Relations of the Medical Profession and Communities, by Robert S. Hamilton, M.D., of Staunton; Supplemental Report on the anatomical, physiological and pathological differences between the White and Black Races, by Thos. P. Atkinson, M.D., of Amelia Co.; On Gun-shot and other wounds of the Peritoneum, by Hunter McGuire, M.D., Professor of Surgery, Med. Col. Va.; On Miasmatic Fevers in Louisa county, by Wm. A. Gillespie, M.D., Louisa county; On Nitrite of Amyl as an antidote to Chloroform, by Wm. C. Dabney, M.D., Charlottesville; On Chloroform in Obstetrical Practice, by A. M. Fauntleroy, M.D., Staunton; Report on Epidemics of Piedmont District, by the Committee; Remarkable recovery from a pistol-shot wound of the right Illium, Pelvis Trephined to drain pyæmic secretion, by S. C. Gleaves, M.D., Wytheville; Report of wound in the Pelvis, by G. Wm. Semple, M.D., Hampton; Saccharated Pepsin, by C. Wesley Thomas, M.D. Norfolk; Some Neglected Points in the pathology of Typhoid Fever, as furnishing the proper indications of treatment, by Samuel K. Jackson, M.D., Norfolk; Reports of cases, Foreign Body in Rectum and Hepatic Abcess, by Wm. H. Bramblett, M.D., Newbern; an improvement on Barnes' and Skene's Uterine Hydrostatic Dilators, by J. St.P. Gibson, M.D., Waynesboro.

---

Report of Wills' Ophthalmic Hospital for the year 1873. Philadelphia, 1874. Octavo; paper, pp. 17. From R. J. Levis, M.D., one of the attending surgeons.

---

Forty-Eighth Annual Report of the Massachusetts Charitable Eye and Ear Infirmary, 1874. Boston: Alfred Mudge & Son Printers, 1874. Octavo; paper, pp. 39.

---

Report of the Vaccine Department of the New York Dispensary, for the year 1873. By Frank P. Foster, M.D. New York: John W. Amerman, Printer, 1874. Octavo; paper, pp. 23.

---

Annual Report of the Board of Health of the City of Pittsburgh, for the year 1873. Pittsburgh, Pennsylvania: Moore & Nesbit, Printers, 1874. Octavo; paper, pp. 95.

